# The Quest for High-Temperature Superconductivity in Nickelates under Ambient Pressure

**DOI:** 10.3390/ma17112546

**Published:** 2024-05-25

**Authors:** Leena Aggarwal, Ivan Božović

**Affiliations:** 1 Brookhaven National Laboratory, Upton, NY 11973, USA; 2 Department of Chemistry, Yale University, New Haven, CT 06520, USA

**Keywords:** high-temperature superconductivity, nickelates, molecular beam epitaxy

## Abstract

Recently, superconductivity with *T_c_* ≈ 80 K was discovered in La_3_Ni_2_O_7_ under extreme hydrostatic pressure (>14 GPa). For practical applications, we needed to stabilize this state at ambient pressure. It was proposed that this could be accomplished by substituting La with Ba. To put this hypothesis to the test, we used the state-of-the-art atomic-layer-by-layer molecular beam epitaxy (ALL-MBE) technique to synthesize (La_1−x_Ba_x_)_3_Ni_2_O_7_ films, varying *x* and the distribution of La (lanthanum) and Ba (barium). Regrettably, none of the compositions we explored could be stabilized epitaxially; the targeted compounds decomposed immediately into a mixture of other phases. So, this path to high-temperature superconductivity in nickelates at ambient pressure does not seem promising.

## 1. Introduction

Arguably, the most disruptive event in the recent history of condensed matter physics was the seminal discovery of high-temperature superconductivity (HTS) in cuprates in 1986 [1], the ripples of which are still being felt. Among others, it triggered a massive quest for other HTS materials. Nickel neighbors Cu in the periodic system; thus, nickelates’ chemistry, crystal structures, and many physical properties resemble those of cuprates. This prompted theorists to speculate that nickelates could also host HTS [2,3,4]. The quest for HTS in nickelates started immediately, but, for over three decades, it has not succeeded. Finally, in 2019, the group led by Harold Hwang at Stanford observed superconductivity with *T_c_* ≈ 8 K in Nd_0.8_Sr_0.2_NiO_2_ [5]. With the focused effort of several groups, this result was improved further by optimizing the synthesis conditions [6,7,8,9,10,11,12]. In La_0.8_Sr_0.2_NiO_2_, the best result reported was *T_c_*^onset^ = 18.8 K and *T_c_* (*R* = 0) = 16.5 K [13]. In 2022, the group at Harvard led by Julia Mundy reported superconductivity in Nd_6_Ni_5_O_12_ with *T_c_*^onset^ ≈ 13 K [14]. Note that both materials contain RNiO_2_ blocks, with R = La or Nd, in which the apical oxygen is removed by “soft chemistry”—the topotactic reduction of the perovskite RNiO_3_ blocks within the precursor material. To accomplish this, the films are annealed in a hydrogen atmosphere or co-annealed in a vacuum with CaH_2_ at a relatively low temperature (250–300 °C), at which the integrity of the NiO_2_ planes is preserved. These results were met with much interest and follow-up research, but *T_c_* stayed low, and the mechanism remained unclear.

However, several months ago, superconductivity in La_3_Ni_2_O_7_ was reported with *T_c_* ≈ 80 K, albeit only under very high hydrostatic pressure (*p* = 18 GPa) [15,16]. This discovery caused much excitement and a flood of new papers, which, so far, have been largely theoretical. However, the basic physics questions about the nature of the HTS state, the order parameter’s symmetry, the pairing mechanism, etc., are all still open and hotly debated. From the practical viewpoint, the central problem is stabilizing the HTS state at ambient pressure, a prerequisite for any application.

**Figure 1:**
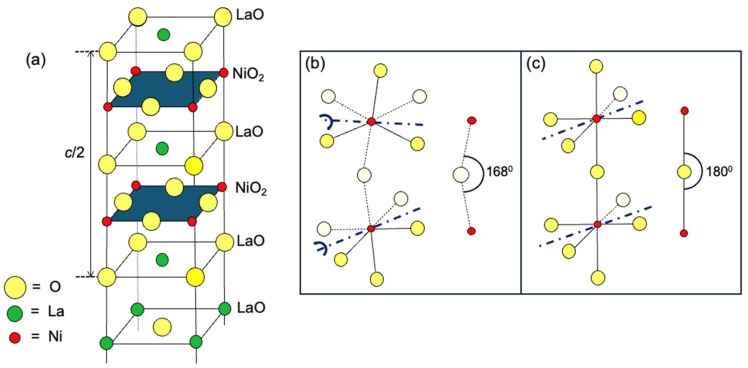
(**a**) The schematic of the La_3_Ni_2_O_7_ structure. Yellow circles denote O, green circles La, and red circles Ni atoms. (**b**) Zoom-in: A buckled NiO_2_ layer with tilted octahedra. White circles are O atoms behind the *xz* plane. (**c**) A straight NiO_2_ layer. The dark blue dash-dot lines are the axis of rotation (buckling).

A hint at where to look may be found in the experimental observation that this HTS state is susceptible to small changes in the crystal structure of La_3_Ni_2_O_7_, illustrated schematically in Figure 1. An idealized structure, with all Ni-O-O bond angles at 180° and all the atoms in the NiO_2_ building block lying in the same plane, is depicted in Figure 1a. A simplified version, showing only two apex-sharing NiO_6_ octahedra, is shown in Figure 1b. This structure hosts HTS under a high pressure. However, in La_3_Ni_2_O_7_ at ambient pressure, the NiO_6_ octahedra are slightly tilted, i.e., rotated by about 6^0^ around the axis parallel to the bisectrix of the octahedron base (a line bisecting the angle formed by two in-plane Ni-O bonds, as marked by a dark blue–dot line in Figure 1b). Two NiO_6_ octahedra sharing the same corner O, apical or equatorial, tilt out-of-phase, one by +6° and the other by −6°. Consequently, the Ni-O_apical_-Ni bond angle decreases to θ_c_ = 168° (see Figure 1b), and the NiO_2_ layers become buckled.

As the hydrostatic pressure is applied and increased, at about 18 GPa, a structural phase transition occurs from the buckled structure to a planar structure. According to [15], the buckled structure has *Cmcm* symmetry and the planar one *Fmmm* symmetry. *Fmmm* symmetry refers to the crystallographic space group #69. The full symbol in the Hermann–Mauguin notation is *F 2/m 2/m 2/m*; the short one is *Fmmm*. It belongs to the orthorhombic crystal class, with the point group **D_2h_** in the Schoenflies notation. It contains three orthogonal order-two rotation symmetry axes, viz. C_2_^a^, C_2_^b^, and C_2_^c^ rotations around the **a**-, **b**-, and **c**-axes, and three mirror-symmetry planes s_a_, s_b_, and s_c_ perpendicular to the **a**-, **b**-, and **c**-axes, respectively. Thus, the Fmmm space group contains (C_2_^a^|0,0,0), (C_2_^b^|0,0,0), (C_2_^c^|0,0,0), (s_c_|0,0,0), (s_a_|0,0,0), (s_b_|0,0,0), and the (E|n_1_, n_2_, n_3_) translations by **t** = n_1_**a** + n_2_**b** + n_3_**c**, as well as all their group products [17]. *Cmcm* symmetry refers to the crystallographic space group #63. (It is sometime referred to also as Amam, which is equivalent, just rotated.) This group also belongs to the orthorhombic crystal class, with point group **D_2h_**. The full Hermann–Mauguin symbol is C 2/m 2/a 2_1_/m. The *Cmcm* space group contains (C_2_^a^|0,0,0), (C_2_^b^|0,0,0), (C_2_^c^|0,0,1/2), (s_a_|0,0,0), (s_b_|0,0,1/2), (s_c_|0,0,0), the (E|n_1_, n_2_, n_3_) translations, and all their group products [17]. 

*Fmmm* and *Cmcm* symmetry refer to the crystallographic space groups #69 and #63, respectively. Both groups belong to the orthorhombic crystal class, with point group **D_2h_** in the Schoenflies notation. The full symbol of the *Fmmm* phase in the Hermann–Mauguin notation is *F 2/m 2/m 2/m*. It contains three orthogonal order-two rotation symmetry axes, viz. C_2_^a^, C_2_^b^, and C_2_^c^ rotations around the **a**-, **b**-, and **c**-axes, and three mirror-symmetry planes s_a_, s_b_, and s_c_ perpendicular to the **a**-, **b**-, and **c**-axes, respectively. Thus, the *Fmmm* space group contains (C_2_^a^|0,0,0), (C_2_^b^|0,0,0), (C_2_^c^|0,0,0), (s_c_|0,0,0), (s_a_|0,0,0), (s_b_|0,0,0), and the (E|n_1_,n_2_,n_3_) translations by **t** = n_1_**a** + n_2_**b** + n_3_**c**, as well as all their group products [17]. The full Hermann–Mauguin symbol of *Cmcm* is *C* 2/m 2/a 2_1_/m. It contains (C_2_^a^|0,0,0), (C_2_^b^|0,0,0), (C_2_^c^|0,0,1/2), (s_a_|0,0,0), (s_b_|0,0,1/2), (s_c_|0,0,0), and (E|n_1_, n_2_, n_3_) translations and all their group products [17]. *Cmcm* is also sometime referred as *Amam*, which is equivalent, just rotated.

Notably, the HTS state emerges concomitantly with this structural transition. This fact has led to speculations that the route to stabilizing the HTS state in La_3_Ni_2_O_7_ at ambient pressure is to suppress this lattice distortion. The critical question is how to achieve this experimentally.

A theoretical prediction was recently posted by Rhodes and Wahl that the *Fmmm* structure may be stabilized in the *n* = 2 RP layered-perovskite structure if La is replaced with larger cations, Ba or Ac (Actinium), exerting intrinsic “chemical” pressure [18]. The rationale is that replacing La with Ac, which is isovalent but has a larger ionic radius, can change the crystal structure to *Fmmm*. At the same time, the electronic states near the Fermi energy (*E_F_*), primarily comprising the Ni 3d and O 2p orbitals, should change very little. If one replaces La^3+^ with Ba^2+^, one expects a more significant change, including a major *E_F_* shift. Rhodes and Wahl performed density functional theory (DFT) calculations to quantify these expectations and explored structural relaxations to determine stable crystal structures. While DFT is known not to capture the strong correlation effects, Rhodes and Wahl argued that structural relaxations should be controlled by chemical bonding and electronic states on much larger energy scales. 

The most interesting insight from these numerical experiments is that, in the *Fmmm* phase with straight Ni-O-Ni bonds, the d_x2−y2_ and d_z2_ bands cross at *E_F_*. Once the structure distorts to *Cmcm* with buckled NiO_6_ octahedra, these bands mix, and their crossing is avoided—i.e., a small hybridization (pseudo)gap opens at *E_F_*. We believe that this result may be valid beyond this particular numeric exercise and relevant to understanding the physics of HTS in compressed La_3_Ni_2_O_7_. 

Of the two proposed substituents, Actinium is impractical because it is a highly radioactive emitter of a-particles, challenging to access and handle, and expensive. It is as dangerous as plutonium, and the stringent BNL safety regulations prohibit its handling. The issue is not with the minuscule amount of Ac in the film but with the typical load of material in the Knudsen-cell crucible, which is on the order of 100 g. That amount of Ac would produce about 68,000 Curie, an extremely high radiation level.

Barium is readily available, but the big unknown is whether Ba_3_Ni_2_O_7_ can be synthesized at all. That would require Ni to assume the formal 4+ oxidation state, which is very rare (and quite unstable) in nickel chemistry. Rhodes and Wahl suggested that one could try a partial La→Ba substitution instead [18], but how much would be needed and sufficient was not quantified.

In the present paper, we report putting to the experimental test the following theoretical predictions [18]: (La_1−x_Ba_x_)_3_Ni_2_O_7_ can be synthesized; the NiO_2_ layers will not buckle; and HTS will stabilize at ambient pressure. 

## 2. Methods

Layered nickelates, also known as Ruddledsen-Popper (RP) phases R_n+1_Ni_n_O_3n+1_, where R is a rare-earth atom and *n* = 1, 2, …, are very complex materials; for example, La_3_Ni_2_O_7_ has 12 atoms in the unit cell. The RP phases with *n* > 3, such as the superconducting Nd_6_Ni_5_O_12_, are not even thermodynamically stable and cannot be synthesized by conventional techniques. Moreover, since the enthalpy of the formation of different phases is very close to one another, entropy favors phase mixing; hence, most nickelate samples end up being multiphase, which hampers the discerning of their intrinsic properties. Thanks to our unique atomic-layer-by-layer molecular beam epitaxy (ALL-MBE) equipment for synthesizing and characterizing complex oxides, our group is well-equipped to address these challenges [19]. 

**Figure 2:**
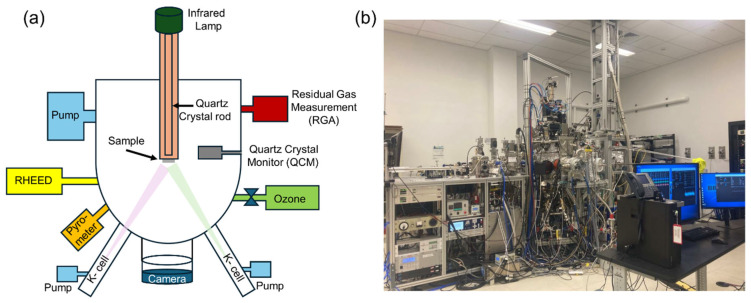
(**a**) The MBE system schematics and (**b**) the MBE system photo.

One of our MBE systems is illustrated in Figure 2. This one has eight thermal-effusion sources (Knudsen-cells) and a pure ozone gas source. The system features our signature modular design, which has been explained in detail before [19]. Each source resides within its autonomous chamber (“arm”), supplied with its turbo-molecular pump, a pneumatically actuated shutter, and a gate valve. Thus, a source can be opened, recharged, serviced, or changed without breaking the ultrahigh vacuum in the main growth chamber, ensuring almost 100% system uptime [19]. The substrate is heated using an infrared lamp and a quartz crystal rod as a waveguide. We coated the backside of every substrate with SrRuO_3_, which is metallic and black, absorbs radiation, is chemically stable, and has a very low vapor pressure, thus providing very uniform substrate heating. The MBE synthesis chamber is equipped with a high-energy electron diffraction (RHEED) reflection system. This MBE synthesis module is connected under ultrahigh vacuum to analytical modules for angle-resolved photoemission spectroscopy (ARPES) and scanning tunneling microscopy (STM) [20,21].

**Figure 3:**
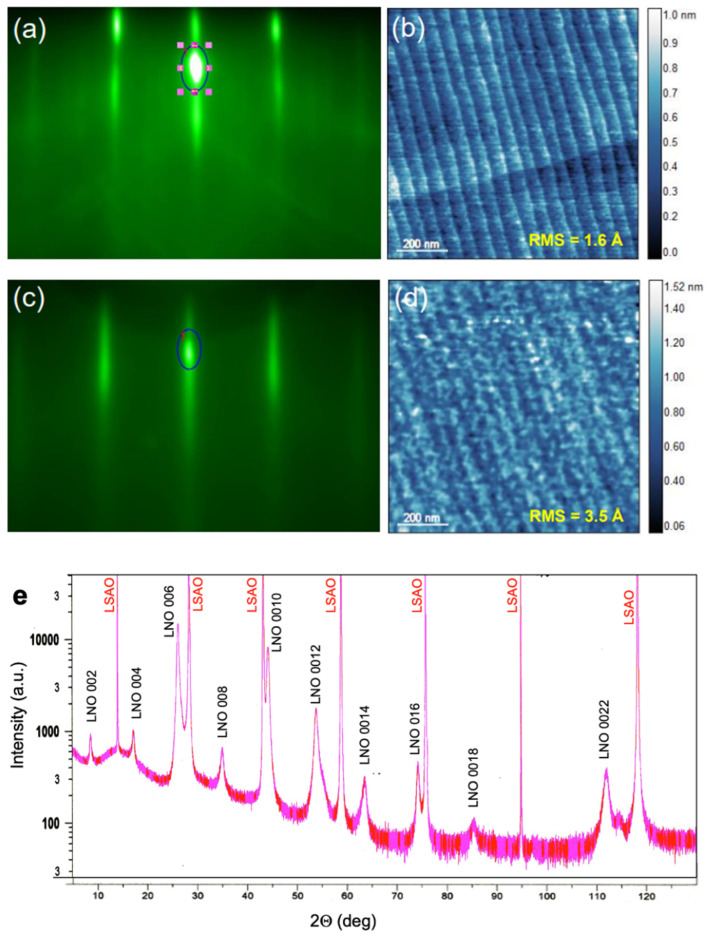
(**a**) RHEED pattern from the STO substrate. The dark blue oval marks the area around the specular reflection spot over which we integrate the intensity and monitor its oscillatory time evolution. (**b**) AFM image of the STO substrate surface. (**c**) RHEED of a La_3_Ni_2_O_7_ film grown on STO. (**d**) AFM of the same La_3_Ni_2_O_7_ film. (**e**) X-ray diffractogram of a La_3_Ni_2_O_7_ film grown on LSAO.

Our nickelate synthesis experiments started with conditioning the substrate surface, which we found is critical to producing high-quality films. We explored single-crystal SrTiO_3_ (STO), Nb-doped SrTiO_3_ (Nb:STO), LaSrAlO_4_ (LSAO), and (LaAlO_3_)_0.3_(Sr_2_TaAlO_6_)_0.7_ (LSAT) substrates polished perpendicular to the [001] crystal axis. STO substrates were prepared by a short etching with buffered HF, after which the surface showed single (TiO_2_) termination. Subsequent annealing at a high temperature (*T* = 1000 °C) improved the substrate surface. Inspection using atomic-force microscopy (AFM) typically showed atomically flat terraces and an RMS surface roughness of 2 Å or even less (Figure 3b). The terrace steps originated from the inevitable substrate miscut from the ideal (001) crystallographic plane. The preparation procedure for LSAO and LSAT did not involve etching, just high-temperature annealing, but we found it critical to place another substrate face-to-face and spaced within a few micrometers to compensate for cation sublimation and loss. The substrate conditioning procedure has been reported in full detail in a previous publication [22].

We used MBE to synthesize La_n+1_Ni_n_O_3n+1_ phases with *n* = 1, 2,…7, the LaNiO_3_ perovskite (frequently referred to as the *n* = ∞ phase), and various heterostructures and superlattices. We explored substituting La with Dy, Y, Sr, and Ce. Of particular interest in this paper, we fabricated single-crystal films of La_3_Ni_2_O_7_ on STO, Nb:STO, LSAO, and LSAT substrates. The typical synthesis conditions were a substrate temperature *T_s_* = 500–750 °C and a background pressure *p* = 1.5 × 10^−6^ to 3 × 10^−5^ Torr of pure ozone. We controlled the kinetics by shuttering. In atomic-layer-by-layer deposition, we deposited one monolayer of a desired metal (La or Ni) at a time. Alternatively, we used block-by-block deposition where the building blocks were LaO and LaNiO_3_ layers; one block of LaO and *n* blocks of LaNiO_3_ were stacked to build one layer of La_n+1_Ni_n_O_3n+1_, which was then repeated. Generally, lower *p*, higher *T_s_*, and block-by-block synthesis resulted in better La_3_Ni_2_O_7_ film morphology. 

After the film’s deposition, we frequently post-annealed the films in situ at a higher ozone pressure (*p* = 1 × 10^−4^ Torr) first at the growth temperature and then at *T_s_* = 300 °C. We also used ex situ annealing in an oxygen–ozone gas mixture with the ozone partial pressure *p* ≈ 50 Torr, at *T_s_* = 200–350 °C. Note that ozone (O_3_) is the second most potent known oxidant, with an oxidation power orders of magnitude larger than that of O_2_, so this procedure is believed to result in backfilling any oxygen vacancies.

Every film was characterized in real-time by RHEED, providing information about the film morphology and crystal structure. Subsequently, the surface morphology was visualized ex situ by atomic-force microscopy (AFM). The typical film projected the terraces and the steps inherited from the substrates and had an RMS surface roughness in the 2–5 Å range. Selected films were also studied by X-ray diffraction (XRD), transport measurements, ARPES, STM, and transmission electron microscopy (TEM); the details will be reported elsewhere.

The principal novelty reported here is the first attempt to wholly or partially substitute La^3+^ by Ba^2+^ in the *n* = 2 nickelate RP phase.

## 3. Results

To test the idea of stabilizing the ambient-pressure HTS state by substituting La in the *n* = 2 RP La_3_Ni_2_O_7_ nickelate structure with Ba, we grew several (La_1−x_Ba_x_)_3_Ni_2_O_7_ films, varying *x* and the distribution of La and Ba. The STO substrate preparation and characterization followed the recipe described in the “Methods”, in Section 2. In Figure 3a, we show the RHEED pattern and, in Figure 3b, the AFM image of the substrate surface before growth. Both are characteristic of an atomically flat STO(001) crystal surface with single (TiO_2_) termination.

To verify that we were using the optimal growth conditions, we first synthesized several single-crystal La_3_Ni_2_O_7_ films on 10 mm × 5 mm × 1 mmSTO and LSAO substrates. In parallel, we used 10 mm × 10 mm × 1 mm STO and LSAO substrates in another ALL-MBE system to synthesize various Ruddledsen-Popper (RP) phases La_n+1_Ni_n_O_3n+1_, with *n* = 1, 2,…, 7. The two MBE systems ran under the same conditions (*T*, *p*, composition, deposition sequence), produced similar results. We used *p* = 1.5 × 10^−6^ Torr of ozone, *T_s_* = 650 °C, and block-by-block deposition sequencing; these choices provided the best morphology for La_3_Ni_2_O_7_. We derived the chemical composition of our films from the absolute rate calibration of our sources, which was accurate to within a couple of %. These were determined by a quartz oscillator rate monitor (QCM) before each synthesis experiment and occasionally double-checked by Rutherford back-scattering (RBS) spectroscopy. RHEED oscillations also provided a convenient method to calibrate the absolute depiction rates of La, Sr, and Ba. Other MBE groups in the field used the same methodology.

Figure 3c shows the RHEED pattern of a high-quality La_3_Ni_2_O_7_ film on the STO(100) substrate. The strong specular reflection, the pronounced oscillations of its intensity as a function of time, the absence of any transmission spots, prominent Kikuchi lines, etc., all indicate single-crystal film growth with an atomically smooth surface. In Figure 3d, we reproduce an AFM image of the surface of the same film, showing that the RMS surface roughness is less than 4 Å, with the steps and terraces projected from the substrate. We note that the feedback we obtain from RHEED (in real time) and AFM (ex situ) is quite informative, because even a minor deviation from the targeted stoichiometry leads to the nucleation of secondary-phase defects, such as 3D outgrowths of NiO or La_2_O_3_, which we can observe even well below 1% abundance. The surface is atomically smooth only when the stoichiometry is precisely correct. A detailed explanation of how this works and can be utilized to make real-time corrections to the growth “recipe” had been published previously for one example Ruddledsen-Popper phase [23].

In Figure 3e, we show an X-ray diffraction pattern obtained from a La_3_Ni_2_O_7_ film deposited on an LSAO substrate. Apart from the Bragg reflections from the substrate and the La_3_Ni_2_O_7_ film, no traces of other phases are noticeable. The *c*-axis lattice constant inferred using the standard Nelson-Riley fitting procedure is 20.64044(6) Å, in good agreement with the literature. (The small differences are likely attributable to variations in the exact oxygen stoichiometry).

Turning to the (La_1−x_Ba_x_)_3_Ni_2_O_7_ film’s synthesis, we started the experiments by depositing an ultrathin (one-unit-cell-thick) layer of La_3_Ni_2_O_7_ to ensure single-crystal film nucleation. We observed perfect RHEED images during and after this buffer layer, essentially identical to that shown in Figure 3c. The successful growth of the La_3_NiO_7_ buffer layer as the template for the subsequent growth of (La_1−x_Ba_x_)_3_Ni_2_O_7_ is a crucial logical check, since any failed outcome, such as defect nucleation, phase separation, film decomposition, etc., cannot be attributed to external factors such as imperfections of the substrate surface, the improper choice of *p*, *T_s_*, or the growth kinetics.

**Figure 4:**
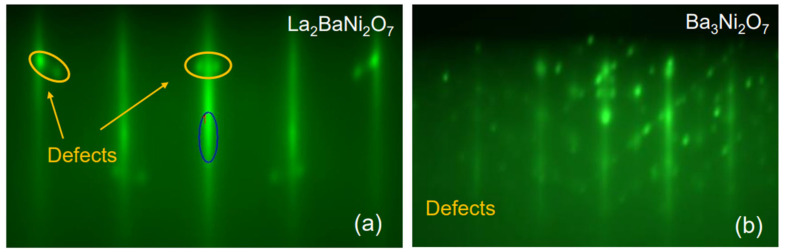
RHEED patterns were recorded after the deposition of a one-unit-cell-thick layer of (**a**) (La_0.5_Ba_0.5_)_3_Ni_2_O_7_ and (**b**) Ba_3_Ni_2_O_7_. The dark blue oval indicates the specular reflection and the yellow ovals indicate the diffraction from NiO defects.

Nevertheless, our attempts to grow (La_2_Ba_0.5_)_3_Ni_2_O_7_ failed. After the first (La_0.5_Ba_0.5_)_3_Ni_2_O_7_ layer, in addition to the RHEED streaks characteristic of the epitaxial *n* = 2 RP nickelate phase, we observed some transmission spots indicating three-dimensional (3D) growth of small precipitates of some unwanted secondary phase (Figure 4a). These transmission spots became prominent after the second (La_0.5_Ba_0.5_)_3_Ni_2_O_7_ layer. To probe the chemical composition of these precipitates, we tried to dissolve them by dosing small amounts of Ni or La to the surface. As we added Ni, the defect-related spots grew stronger. When we added LaO, they weakened and eventually disappeared, indicating that the precipitates were dissolved or buried. We inferred that these diffraction spots originated from the formation of 3D islands of NiO, sticking out of the film surface enough to allow electron transmission. Note that the lattice constant of NiO is *a* ≈ 4.3 Å, about 10% larger than that of STO (*a* = 3.905 Å). Since RHEED images are mapping the Bragg diffraction features in the reciprocal space, one would expect the NiO-related spots to appear at about 10% on the inside of the first-order RHEED streaks of La_3_Ni_2_O_7_. This is consistent with what is seen in Figure 4a. With the spot size roughly an order of magnitude smaller than the separation between the zeroth and first-order reflection streaks, we roughly estimated the island size to be in the 50–100 Å range.

Given the above, we suggest that we probably induced the following chemical reaction:

(1) $$2{\left({\mathrm{La}}_{0.5}{\mathrm{Ba}}_{0.5}\right)}_{3}{\mathrm{Ni}}_{2}O_{7}\;\to \;3{\left({\mathrm{La}}_{0.5}{\mathrm{Ba}}_{0.5}\right)}_{2}{\mathrm{NiO}}_{4}+\mathrm{NiO}\;+0.5O_{2}\;$$

Our attempts to grow La_2_BaNi_2_O_7_ produced a similar outcome, i.e., immediate nucleation of 3D islands of NiO; the resulting RHEED pattern was identical to that shown in Figure 4a. The probable reaction here was the following:

(2) $$2{\mathrm{La}}_{2}{\mathrm{BaNi}}_{2}O_{7}\;\to \;3{\left({\mathrm{La}}_{0.67}{\mathrm{Ba}}_{0.33}\right)}_{2}{\mathrm{NiO}}_{4}+\mathrm{NiO}\;+0.5O_{2}\;$$

In (1) and (2) above, we assume that LaBaNiO_4_ and La_1.33_Ba_0.67_NiO_4_ are stable or epitaxially stabilized. Indeed, it was shown earlier that Ba is soluble in La_2_NiO_4_. Crystals of (La_1−x_Ba_x_)_2_NiO_4_ have been synthesized with *x* ≤ 1 [24,25]. This is still allowed by nickel chemistry since, e.g., in LaBaNiO_4_, the formal valence of Ni is 3+, which is still accessible, while a valence larger than 3+ is not, at least not under the standard conditions. LaBaNiO_4_ is very insulating. It is tetragonal (*I4/mmm*) with the in-plane lattice constant reported as *a* = 3.9013 Å [21] or 3.8552 Å [25], in either case very close to that of STO. We have in fact already succeeded in synthesizing thin films of LaBaNiO_4_, as well as several other La_1+x_Ba_1−x_NiO_4_ phases, on STO. This provides some additional experimental support to our hypotheses formulated in (1) and (2) above. However, we leave the details to a separate future report since, here, our focus is on the La_3_Ni_2_O_7_ structure that hosts HTS under extreme pressures and has been predicted to achieve it at ambient pressure upon Ba-La substitution.

When we tried Ba_3_NiO_7_, the outcome was dramatically different (worse) (see Figure 4b). The surface was immediately covered with small crystallites of some compound, growing in 3D and in a strange orientation (tilted by about 45° with respect to the substrate (001) facet). The exact chemical composition and crystal structure of this unwanted phase have not been determined at this time. If we follow the reasoning of (1) and (2), we infer that Ba_2_NiO_4_ is probably unstable itself; symptomatically, we could not find any reference in the literature to its being synthesized. 

Nevertheless, the grand total is clear: Ba_3_Ni_2_O_7_ is extremely unstable, and it decomposes instantaneously, at least under our synthesis conditions (which we proved are quite favorable for the growth of La_3_Ni_2_O_7_ and other La-based RP nickelate phases, with *n* = 1 to 7). The partial substitution of Ba with La produces 3D islands of a secondary phase, most likely NiO, embedded within a flat, epitaxial layered nickelate matrix, most likely with the *n* =1 RP crystal structure. 

We have yet to explore doping La_3_Ni_2_O_7_ with much smaller doses of Ba. In principle, one may expect that (La_1−x_Ba_x_)_3_Ni_2_O_7_ films could be grown with a very low Ba doping level x, say 5% or less. However, the small perturbation caused by trace amounts of Ba is unlikely to accomplish the desired effect of suppressing the buckling distortion of the NiO_2_ layers. This would defeat the purpose, which is to induce a structural phase transition to the *Fmmm* phase and stabilize flat NiO_2_ planes. Given the experiments’ complexity and costs, this should first be studied theoretically and quantified. A precise prediction should be made about the minimum Ba doping level sufficient to make NiO_2_ layers flat, which could then be tested experimentally. However, we are not very optimistic about the prospects, since, as we have seen, the predictions fell short even for Ba_3_Ni_2_O_7_.

## 4. Conclusions and Outlook

Our main result is that, experimentally, Ba_3_Ni_2_O_7_, (La_0.5_Ba_0.5_)_3_NiO_7_, and La_2_BaNi_2_O_7_ are very unstable. Not even a 1UC thick layer can be epitaxially stabilized; the decomposition is immediate, likely to an R_2_NiO_4_ phase with R = La, Ba (which keeps growing epitaxially), and NiO (which forms 3D islands). Thus, regrettably, substituting La with Ba in R_3_Ni_2_O_7_ does not seem a very promising route to stabilize the HTS state at ambient pressure.

This raises the question of why the theoretical prediction made in Reference [18] failed. One possibility is that this is related to the known DFT’s inability to adequately describe the ground state of strongly correlated electron materials, of which nickelates and cuprates are prime examples—e.g., DFT predicts La_2_CuO_4_ to be metallic, while, experimentally, it is an antiferromagnetic insulator. In nickelates, some relevant electron energy bands near the Fermi level are strongly renormalized; the DFT bands are as much as 500–800% wider than the bands measured by ARPES experiments [26,27,28]. Rhodes and Wahl have argued that the chemistry and crystal structure are usually controlled by the electron spectrum and states at higher (few eV) energy scales and that DFT adequately describes these [18]. Are nickelates unusual in this respect? Or, perhaps, not all relaxation (e.g., decomposition) channels were explored. Our experimental results could motivate theorists to investigate the decomposition routes described by Equations (1) and (2) and compare the total energies of the left- and right-hand compounds. From the chemistry viewpoint, the 4+ nickel oxidation state is extremely rare, and the few known example compounds are quite unstable. As for the physical constraints, one should be aware of the limits of the internal strain tolerance. 

It may be prudent to point out some limitations of our experimental study. One is that we explored a finite range of synthesis conditions (*T*, *p*, composition, deposition sequence). However, it seems unlikely that drifting far out of these ranges would help. We obtained polycrystalline or amorphous films at low *T* and high *p*, even for La_3_Ni_2_O_7_, without any Ba. At too-high *T* and -low *p*, the La_3_Ni_2_O_7_ compound decomposes. It has been shown in Reference [29] that, at high *T*, La_3_Ni_2_O_7_ transforms into La_2_NiO_4_; this is analogous to and consistent with Equations (1) and (2). 

The other limitation is that we did not explore all the possible values of *x* in (La_1−x_Ba_x_)_3_Ni_2_O_7_, but just three representative ones (100%, 50%, and 33%). However, since all three decomposed, it is improbable that some other composition in the range of 33% < x < 100% would be stable. On the low side, the film may grow well for *x* < 5% or so. However, it is unlikely to accomplish what is wanted, i.e., flatten the NiO_2_ planes and, thus, would not be of high interest in the context of stabilizing high-temperature superconductivity in nickelates at ambient pressure.

Looking to the future, the question is what else can be tried. First, one could study the (La_1−x_Ba_x_)_3_Ni_2_O_7_ compounds some more, particularly exploring different thermodynamic (*T*, *p*), kinetic, and epitaxial conditions (i.e., other substrates and facet orientations). The caveat is that this path is time-consuming and not promising. Theoretical guidance would help narrow down the search space, but the problem is that predicting which choice of deposition kinetics will freeze metastable states is a difficult task. Since one wants to raise the Ni oxidation state towards 4+, a more promising experimental approach—regrettably, not available to us—may be to attempt synthesizing Ba_3_Ni_2_O_7_ under extreme oxygen pressure and quenching it to the ambient pressure [30]. 

An alternative path, amenable to our ALL-MBE synthesis technique, is to explore other RP and reduced-RP nickelate phases with different Ni oxidation states. In this case, the band structure details will differ from those in the compressed La_3_Ni_2_O_7_ that hosts HTS. But, we could try doping (by various chemical substitutions, annealing in ozone or vacuum, or electrolyte gating), epitaxial and uniaxial pressure, etc., to tune *E_F_* to the peak in the density of states that originates from a flat band. This task may be challenging, but the impetus is very high.

## Data Availability

The original contributions presented in the study are included in the article, further inquiries can be directed to the corresponding author.

## References

[1] Bednorz G., Müller K.A. (1986). Possible high T_c_ superconductivity in the Ba-La-Cu-O system. Z. Phys. B.

[2] Anisimov V.I., Bukhvalov D., Rice T.M. (1999). Electronic structure of possible nickelate analogs to the cuprates. Phys. Rev. B.

[3] Chaloupka J., Khaliullin G. (2008). Orbital order and possible superconductivity in LaNiO_3_/LaMO_3_ superlattices. Phys. Rev. Lett..

[4] Poltavets V.V., Greenblatt M., Fecher G.H., Felser C. (2009). Electronic Properties, Band Structure, and Fermi Surface Instabilities of Ni^1+^/Ni^2+^ Nickelate La_3_Ni_2_O_6_, Isoelectronic with Superconducting Cuprates. Phys. Rev. Lett..

[5] Li D., Lee K., Wang B.Y., Osada M., Crossley S., Lee H.R., Cui Y., Hikita Y., Hwang H.Y. (2019). Superconductivity in an infinite-layer nickelate. Nature.

[6] Zeng S., Tang C.S., Yin X., Li C., Li M., Huang Z., Hu J., Liu W., Omar G.J., Jani H. (2020). Phase diagram and superconducting dome of infinite-layer Nd_1−x_Sr_x_NiO_2_ thin films. Phys. Rev. Lett..

[7] Lee K., Goodge B.H., Li D., Osada M., Wang B.Y., Cui Y., Kourkoutis L.F., Hwang H.Y. (2020). Aspects of the synthesis of thin film superconducting infinite-layer nickelates. APL Mater..

[8] Li D., Wang B.Y., Lee K., Osada M., Harvey S.P., Kourkoutis L.F., Hwang H.Y. (2020). Superconducting Dome in Nd_1−x_Sr_x_NiO_2_ Infinite Layer Films. Phys. Rev. Lett..

[9] Hepting M., Li D., Jia C.J., Lu H., Paris E., Tseng Y., Feng X., Osada M., Been E., Hikita Y. (2020). Electronic structure of the parent compound of superconducting infinite-layer nickelates. Nat. Mater..

[10] Goodge B.H., Li D., Lee K., Osada M., Wang B.Y., Sawatzky G.A., Hwang H.Y., Kourkoutis L.F. (2021). Doping evolution of the Mott–Hubbard landscape in infinite-layer nickelates. Proc. Natl. Acad. Sci. USA.

[11] Yang C., Wang Y., Putzky D., Sigle W., Wang H., Ortiz R.A., Logvenov G., Benckiser E., Keimer B., Van Aken P.A. (2022). Ruddlesden–Popper Faults in NdNiO_3_ Thin Films. Symmetry.

[12] Fürsich K., Pons R., Bluschke M., Ortiz R.A., Wintz S., Schierle E., Weigand M., Logvenov G., Schütz G., Keimer B. (2022). Oxygen Hole Character and Lateral Homogeneity in PrNiO_2+d_ Thin Films. Front. Phys..

[13] Sun W., Li Y., Liu R., Yang J., Li J., Wei W., Jin G., Yan S., Sun H., Guo W. (2023). Evidence for Anisotropic Superconductivity Beyond Pauli Limit in Infinite-Layer Lanthanum Nickelates. Adv. Mater..

[14] Pan G.A., Segedin D.F., LaBollita H., Song Q., Nica E.M., Goodge B.H., Pierce A.T., Doyle S., Novakov S., Carrizales D.C. (2022). Superconductivity in a quintuple-layer square-planar nickelate. Nat. Mater..

[15] Sun H., Huo M., Hu X., Li J., Han Y., Tang L., Mao Z., Yang P., Wang B., Cheng J. (2023). Signatures of superconductivity near 80 K in a nickelate under high pressure. Nature.

[16] Wang G., Wang N.N., Shen X.L., Hou J., Ma L., Shi L.F., Ren Z.A., Gu Y.D., Ma H.M., Yang P.T. (2024). Pressure-Induced Superconductivity in Polycrystalline La_3_Ni_2_O_7−δ_. Phys. Rev. X.

[17] Aroyo M.I. (2016). International Tables for Crystallography.

[18] Rhodes L.C., Wahl P. (2024). Structural routes to stabilize superconducting La_3_Ni_2_O_7_ at ambient pressure. Phys. Rev. Mater..

[19] Bozovic I. (2001). Atomic-Layer Engineering of Superconducting Oxides: Yesterday, Today, Tomorrow. IEEE Trans. Appl. Superconduct..

[20] Wu Z., Putzky D., Kundu A.K., Li H., Yang S., Du Z., Joo S.H., Lee J., Zhu Y., Logvenov G. (2020). A homogeneous superconducting gap in DyBa_2_Cu_3_O_7−δ_ synthesized by oxide molecular beam epitaxy. Phys. Rev. Mater..

[21] Kim C.K., Drozdov I., Fujita K., Davis J.C., Božović I., Valla T. (2022). ⁠ In-situ angle-resolved photoemission spectroscopy of copper-oxide thin films synthesized by molecular beam epitaxy. J. Electron Spectrosc..

[22] He X., Xu X., Shi X., Božović I. (2023). Optimization of La_2−x_S_rx_CuO_4_ Single Crystal Film Growth via Molecular Beam Epitaxy. Condens. Matter.

[23] Božović I., Eckstein J.N. (1995). Real-time, in-situ Study of Growth of Complex Oxides by RHEED. MRS Bull..

[24] Alonso J.A., Amador J., Gutierrez-Puebla E., Monge M.A., Rasines I., Ruiz-Valero C., Campa J.A. (1990). Persistence of the La_2_NiO_4_ crystal structure in La_2−x_Ba_x_NiO_4_ samples with high Ba contents (x < 1). Solid State Commun..

[25] Schilling A., Dell’Amore R., Karpinski J., Bukowski Z., Medarde M., Pomjakushina E., Mueller K.A. (2009). LaBaNiO_4_: A Fermi glass. J. Phys. Condens. Matter.

[26] Yang J., Sun H., Hu X., Xie Y., Miao T., Luo H., Chen H., Liang B., Zhu W., Qu G. (2023). Orbital-Dependent Electron Correlation in Double-Layer Nickelate La_3_Ni_2_O_7_. arXiv.

[27] Abadi S.N., Xu K.-J., Lomeli E.G., Puphal P., Isobe M., Zhong Y., Fedorov A.V., Mo S.-K., Hashimoto M., Lu D.-H. (2024). Electronic structure of the alternating monolayer-trilayer phase of La_3_Ni_2_O_7_. arXiv.

[28] Li H., Hao P., Zhang J., Gordon K., Linn A.G., Chen X., Zheng H., Zhou X., Mitchell J.F., Dessau D.S. (2023). Electronic structure and correlations in planar trilayer nickelate Pr_4_Ni_3_O_8_. Sci. Adv..

[29] Gao X., Liu J., Ji X., Wei L., Xiao W., Hu S., Li L., Gan Y., Chen K., Liao Z. (2023). Thermodynamic-driven selective synthesis and phase transformation of Sr-doped neodymium nickelate Ruddlesden-Popper epitaxial films. APL Mater..

[30] Deng L., Bontke T., Dahal R., Xie Y., Gao B., Li X., Yin K., Gooch M., Rolston D., Chen T. (2021). Pressure-induced high-temperature superconductivity retained at ambient. Proc. Natl. Acad. Sci. USA.

